# Stable Iterative Variable Selection

**DOI:** 10.1093/bioinformatics/btab501

**Published:** 2021-07-16

**Authors:** Mehrad Mahmoudian, Mikko S Venäläinen, Riku Klén, Laura L Elo

**Affiliations:** Turku Bioscience Centre, University of Turku and Åbo Akademi University, Turku, Finland; Department of Future Technologies, University of Turku, Turku, Finland; Turku Bioscience Centre, University of Turku and Åbo Akademi University, Turku, Finland; Turku Bioscience Centre, University of Turku and Åbo Akademi University, Turku, Finland; Turku Bioscience Centre, University of Turku and Åbo Akademi University, Turku, Finland; Institute of Biomedicine, University of Turku, Turku, Finland

## Abstract

**Motivation:**

The emergence of datasets with tens of thousands of features, such as high-throughput omics biomedical data, highlights the importance of reducing the feature space into a distilled subset that can truly capture the signal for research and industry by aiding in finding more effective biomarkers for the question in hand. A good feature set also facilitates building robust predictive models with improved interpretability and convergence of the applied method due to the smaller feature space.

**Results:**

Here, we present a robust feature selection method named Stable Iterative Variable Selection (SIVS) and assess its performance over both omics and clinical data types. As a performance assessment metric, we compared the number and goodness of the selected feature using SIVS to those selected by Least Absolute Shrinkage and Selection Operator regression. The results suggested that the feature space selected by SIVS was, on average, 41% smaller, without having a negative effect on the model performance. A similar result was observed for comparison with Boruta and caret RFE.

**Availability and implementation:**

The method is implemented as an R package under GNU General Public License v3.0 and is accessible via Comprehensive R Archive Network (CRAN) via https://cran.r-project.org/package=sivs.

**Supplementary information:**

Supplementary data are available at *Bioinformatics* online.

## 1 Introduction

Due to more and more cost-efficient data generation and collection methods, we have seen a substantial rise in the data volume of database submissions during the past decade. To put it in perspective, only in the Gene Expression Omnibus (GEO) database alone, there has been about a 9-fold increase in the number of omics datasets submitted from 2009 to 2019 compared to its preceding decade (1999–2009). The complexity of biological data and the high-dimensionality of the datasets impose a challenge in the analysis and interpretation of these datasets (Braun, 2014). Furthermore, with the current pace of technological advancements, we are getting more and more measurable features to be added to enrich our datasets, which leads to a constant increase in the dimensionality of the feature space. All these make it crucial to find the most effective and influential features from the feature spaces in order to reduce the number of measured features, which ultimately will reduce the data collection costs. On top of reducing the feature space, it is of utter importance to have a robust set of markers and models that are generalizable to other datasets beyond those that were used to train the models.

Feature selection is a crucial part of machine learning in which the features that are most informative in relation to the response value are selected, while irrelevant and redundant features are discarded (Koller and Sahami, 1996; Lin *et al.*, 2014). One of the commonly used methods for high-dimensional data is generalized linear modeling in combination with a shrinkage method, namely Least Absolute Shrinkage and Selection Operator (LASSO) or Elastic Net (Tibshirani, 1996; Zou and Hastie, 2005), which efficiently reduces the feature space and also provides easily interpretable models. However, the major drawback of these methods is inconsistencies in the selected features and their number (Roberts and Nowak, 2014). This is mainly due to hyperparameter tuning that happens via cross-validation. Because of the nature of cross-validation, the resulting models are sensitive to the fold assignment causing inconsistencies between features obtained from multiple runs. Furthermore, in high-dimensional data, the massive difference between the feature space size versus the sample size can further increase this inconsistency. This, in turn, can drastically reduce the reproducibility of the study and cause vast disagreement between studies that have used the same or similar data and yet derived different conclusions and set of selected biomarkers, for example.

LASSO and Elastic Net both fall into the category of embedded feature selection approaches in which the feature selection is made as a part of the classification algorithm (He and Yu, 2010; Mahendran *et al.*, 2020; Wang *et al.*, 2016). Recently, novel hybrid approaches have also been proposed that take advantage of multiple feature selection strategies, such as the conventional filter and wrapper approaches (Apolloni *et al.*, 2016). Despite their good applicability and improved performance in high-dimensional data compared to conventional feature selection algorithms (Lu *et al.*, 2017; Wei *et al.*, 2020), there is a need for novel, robust approaches that have a publicly available implementation that any researcher can easily apply to their own datasets.

In this paper, we present a feature selection method, Stable Iterative Variable Selection (SIVS), and its implementation in R to effectively reduce the feature space to a small subset without decreasing the accuracy. This is achieved by considering multiple configurations of cross-validation sample binning, aggregation of the results for feature ranking, and ultimately shrinking the feature space.

## 2 Materials and methods

**Fig. 1.**
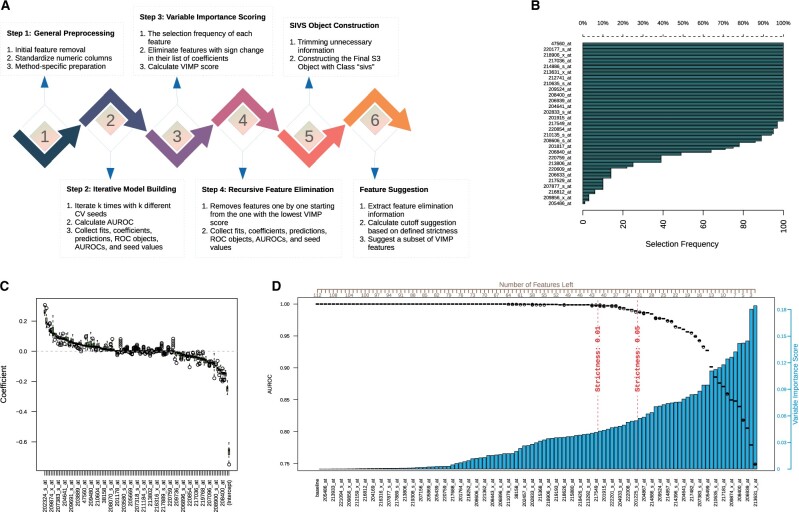
Internal steps of SIVS method. (**A**) The general schema of the SIVS method. (**B**) Frequency of each feature having nonzero coefficient in the ‘iterative model building’ step. (**C**) Distribution of nonzero coefficients each feature has got in the ‘iterative model building’ step. Features are illustrated in a sorted order based on the median of their nonzero coefficients from high to low. (**D**) The main plot of the SIVS method, presenting an overview of the ‘RFE’ step. This plot is composed of three main elements: the bar chart that shows the VIMP, the box plots to show the distribution of AUROC after removal of each feature, and ultimately the two vertical dashed lines marking the two suggested strictness

The general idea of SIVS is to encapsulate methods with embedded feature selection that are not robust in converging to the same feature space, thus resulting in inconsistent model performance. This is done via performing model construction multiple times and aggregating the resulting selected features. By repeatedly constructing models using different cross-validation sample binnings, we ensure that we have covered most, if not all, sample-binning compositions. The overall workflow of SIVS is summarized in Figure 1A and represented in detail in the following sections. From hereafter, the encapsulated method is referred to as the ‘internal method’. In this article, we focus on using a multivariable Generalized Linear Modeling method implemented in R with LASSO and Elastic-Net regularization (glmnet) (Friedman *et al.*, 2010; Simon *et al.*, 2011) as the internal method, but the general concept can be extended to basically any method with cross-validation-dependent embedded feature selection.

### 3.1 Step 1: general preprocessing

The SIVS algorithm starts with preprocessing of the data, which includes removing redundant features and standardizing numeric values for the following steps. First, the numeric features with zero variance or categorical features containing a single class are removed from the data. Finally, all numeric features are standardized to have a mean of zero and a standard deviation of one. This is to make the models' coefficients comparable.

At this point, we perform method-specific preprocessing if required by the internal method. For instance, glmnet prefers to have the input matrix as a ‘data.matrix’ object. Furthermore, glmnet is sensitive to missing values and, therefore, any sample with missing values is removed. Alternatively, imputation could be applied before applying SIVS by the user to retain more samples in the analysis.

### 3.2 Step 2: iterative model building

At the second step, a predefined number k of models (by default k = 100) are built using different cross-validation binnings and all the resulting models are collected, in addition to their prediction performance against the training data. In the implementation of the SIVS R package, the number of iterations can be configured by the user with regard to the sample size. A relatively high number of iterations would result in having the same binning configuration multiple times and consequently does not provide new information. On the other hand, a relatively low number of iterations would lead to not covering all binning arrangements. In practice, we have observed that 100 iterations is sufficient for obtaining a stable set of features in a range of datasets (Klén *et al.*, 2019, 2020; Venäläinen *et al.*, 2020, 2021).

### 3.3 Step 3: variable importance (VIMP) scoring

Based on the set of models built during the iterative step, VIMP score is calculated for each feature. The main idea is to assign a higher score to features that are selected by the majority of the models and are contributing the most in the model to predict the response value. Let us denote the features by $f_{1},f_{2},\ldots ,f_{n}$, where n > 0 is the total number of features. For each feature $f_{i}^{\;}$, the coefficients of the models built in SIVS are denoted by $c_{i}=\left(c_{i,\;1},c_{i,\;2},\ldots ,c_{i,\;k}\right)$ and the vector of elements of $c_{i}$ with nonzero values is denoted by $c_{i}^{*}$. The VIMP score is calculated by the following equation for features where $c_{i}^{*}$ has a length greater than zero:

$$\mathrm{VIMP}\left(f_{i}\right)=\frac{I\left(c_{i}^{*}\right)\times m_{\mathrm{abs}}\left(c_{i}^{*}\right)\times \left|c_{i}^{*}\right|}{1+\mathrm{IQR}\left(c_{i}^{*}\right)},$$

 

(1) Ici*=1,ci, j*<0 for all j,1,ci, j*>0 for all j,0, otherwise,

where $I\left(c_{i}^{*}\right)$ is a binary value indicating if all elements of $c_{i}^{*}$ are either positive or negative, $m_{\mathrm{abs}}$ is the median of absolute values,   is the number of elements, and IQR is the interquartile range.

### 3.4 Step 4: recursive feature elimination (RFE)

For the last step, all features with nonzero VIMP scores based on Equation (1) are kept in the analysis and fed into the last step, and the rest of the features get eliminated. During the RFE step, the features are removed one by one from the model building in the increasing order of their VIMP score calculated in the previous step. Upon eliminating each feature, a set of models (default = 100) are built using the remaining features and different cross-validation seeds to provide an unbiased view of the effect of the eliminated feature on the overall performance of the model.

In the implementation of the SIVS R package, the output of all the aforementioned steps is returned as an S3 R object. There are also helper functions to assist in the interpretation and plotting of the results.

To assist in choosing an appropriate cutoff for the features on the basis of their decreasing VIMP scores, we have implemented a method using the RFE output in the R package (Fig. 1D). This method has a parameter γ to adjust the strictness of the cutoff suggestion (0<γ<1). The cutoff is calculated according to Equation (2):

(2) $$\mathrm{suggested}.\mathrm{cutoff}=\left[\left(1-\gamma \right)\times \left({\tilde{x}}_{\max}-{\tilde{x}}_{\min}\right)\right]+{\tilde{x}}_{\min}$$

where ${\tilde{x}}_{\min}$ and ${\tilde{x}}_{\max}$ are, respectively, the minimum and maximum of the Area Under the Receiver Operating Characteristic curves (AUROC) across the features in the feature elimination step. This cutoff practically defines the minimum acceptable AUROC over the training set. The higher the strictness parameter γ is, the lower the suggested cutoff gets. By default, γ=0.01 which is considered as loose and retains more features.

### 3.5 Data and test design

To evaluate the performance of SIVS for feature selection, we applied it on three different types of biomedical data in a binary classification setup using glmnet with 10-fold cross-validation and binomial family as the internal method. The goodness of the resulting features was assessed by comparing the models built using features suggested by SIVS, with corresponding models built without SIVS. For each of the training data, 100 different logistic regression models were built using the binomial family of glmnet with different cross-validation random seeds to assess the consistency of the resulting performance in the independent validation data. All used datasets are summarized in Table 1 and described in more detail below.

**Table 1.** Data that has been used in this study

Disease	Response value	Data type (platform)	Accession ID
Breast cancer	Relapse-free survival	Microarray (GPL96)	GSE2034, GSE7390
Lung cancer	Subtype classification	RNA-seq	TCGA_LUAD, TCGA_LUSC
Cardiovascular	Occurrence of cardiovascular outcome	Clinical	SPRINT, ACCORD-BP
Arcene	Cancer versus healthy	Mass-spectrometry	ARCENE

*Note:* To compare the method introduced in this article, four types of data have been used. This table presents the various data types that have been used in this article, in addition to the information on what has been used as response values.

As a performance metric, we used AUROC. To test whether the observed difference in AUROC was significant, we performed a pair-wise AUROC comparison between each of the ROC curves of (1) the standard glmnet model and (2) the glmnet model that was built using SIVS suggested features when the same cross-validation seed was used. The pair-wise statistical comparison was performed using the DeLong method (DeLong *et al.*, 1988) implemented in the roc.test function in the pROC (Robin *et al.*, 2011) package. A list of all used R packages is presented in Table S1 in Appendix.

#### 3.5.1 Breast cancer

For breast cancer classification, we used two gene expression microarray datasets from the GEO database, namely GSE2034 (Wang *et al.*, 2005) and GSE7390 (Desmedt *et al.*, 2007). Both datasets have been generated using the Affymetrix Human Genome U133A Array platform and contained lymph-node negative breast cancer samples and their relapse-free survival information. The GSE2034 data consisted of a total of 286 patients, where 179 were relapse-free, and the other 107 were relapsed patients, whereas the GSE7390 consisted of 107 relapse-free and 91 relapsed patients. The relapse-free status of the patients was used as a binary response value in the analysis. The dataset with the larger sample size, GSE2034, was used as the training set, whereas the GSE7390 data was used for independent validation.

To make both datasets comparable, the microarray datasets were independently preprocessed using the Oligo R package ( Carvalho and Irizarry, 2010) and then independently normalized by variance stabilization (Huber *et al.*, 2002) using vsn R package (Huber *et al.*, 2021).

#### 3.5.2 Lung cancer

For lung cancer classification, we used two RNA-seq datasets from The Cancer Genome Atlas (TCGA) database, namely Lung Adenocarcinoma (TCGA-LUAD) and Lung Squamous Cell Carcinoma (TCGA-LUSC). Both datasets were downloaded in the Fragments Per Kilobase of transcript per Million mapped reads upper quartile (FPKM-UQ) normalized (Bioinformatics Pipeline: mRNA Analysis—GDC Docs; HTSeq-FPKM-UQ—GDC Docs; Shahriyari, 2019) format using the following two queries:

- cases.primary_site in [‘bronchus and lung’] and cases.project.project_id in [‘TCGA-LUAD’] and files.access in [‘open’] and files.analysis.workflow_type in [‘HTSeq—FPKM-UQ’] and files.data_type in [‘Gene Expression Quantification’]
- cases.primary_site in [‘bronchus and lung’] and cases.project.project_id in [‘TCGA-LUSC’] and files.access in [‘open’] and files.analysis.workflow_type in [‘HTSeq—FPKM-UQ’] and files.data_type in [‘Gene Expression Quantification’]

The TCGA-LUAD and TCGA-LUSC contained 594 and 551 samples, respectively. Considering that these two datasets contained samples from different subtypes of Lung cancer, we used their combination and built a model to differentiate the two subtypes. To form the training and validation sets, we randomly selected 100 samples from each subtype to create the validation set and used the rest of the samples (*N* = 945) as the training set.

#### 3.5.3 Cardiovascular disease

For prediction of cardiovascular disease events, we used data from two clinical trials, namely the Systolic Blood Pressure Intervention Trial (SPRINT) and the Action to Control Cardiovascular Risk in Diabetes Blood Pressure (ACCORD-BP) trial, both of which compared two antihypertensive treatment strategies and their effects on cardiovascular outcomes (Buse, 2007; Wright *et al.*, 2015). The SPRINT and ACCORD-BP datasets involved 9361 and 4733 participants, respectively. Here, we applied SIVS on SPRINT data to predict the occurrence of primary composite cardiovascular disease outcome (the first occurrence of myocardial infarction, acute coronary syndrome, stroke, heart failure or death from cardiovascular causes) and validated the performance of the models against ACCORD-BP data. Both datasets were available on request from the National Heart, Lung and Blood Institute's (NHLBI) Biologic Specimen and Data Repository Information Coordinating Center (BioLINCC, https://biolincc.nhlbi.nih.gov/). Here, we used similar variable preprocessing as described before (Venäläinen *et al.*, 2020).

#### 3.5.4 Arcene data

In addition to the datasets above, we used a publicly available benchmarking dataset. This dataset is a benchmarking dataset made by aggregating three different mass-spectrometry datasets, and has been designed for testing the performance of feature selection methods and has been used in the NIPS 2003 feature selection challenge (Guyon *et al.*, 2005). This dataset has 1000 anonymized features and separate training set and validation set, each with 100 anonymized samples. Both the training and validation set have 44 samples with a positive response and 56 samples with a negative response.

#### 3.5.5 Comparison to available feature selection methods

We compared the performance of SIVS against two publicly available, widely used feature selection methods: Boruta and RFE. Boruta is an iterative feature selection algorithm based on the random forest classification algorithm (Kursa and Rudnicki, 2010). In each iteration, it uses shuffled shadow variables and calculates *Z* scores to determine feature importance. RFE is a feature ranking method, which starts from a model with all features and in each iteration drops out a certain number of least important features (Guyon *et al.*, 2002). For these methods, we used implementations available in R packages *Boruta* and *caret*, respectively. RFE could only be applied to cardiovascular disease and Arcene datasets due to memory issues occurring with the handling of RNA-seq and microarray data with over 20 000 variables.

## 3 Results

**Fig. 2.**
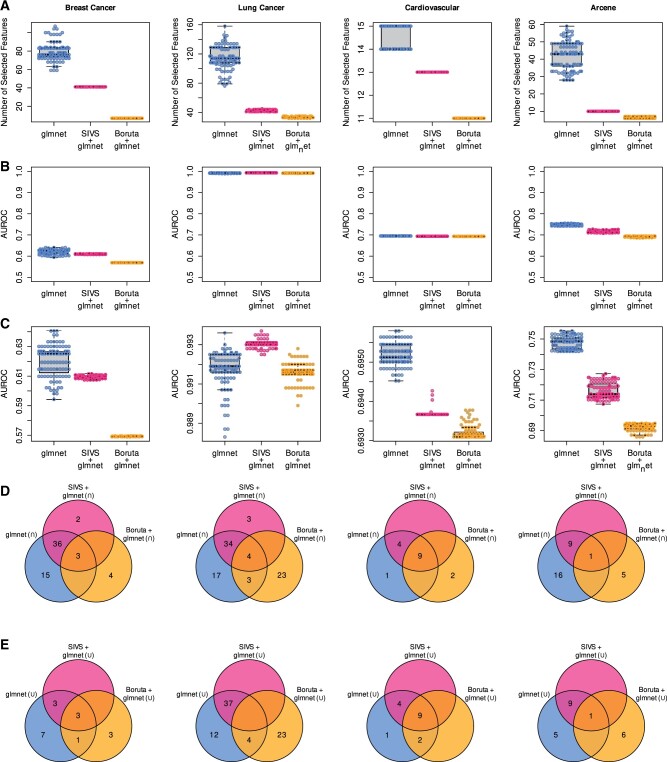
Side-by-side comparison of glmnet and SIVS. (**A**) The number of features that were used in each of the 100 glmnet models built using SIVS features (SIVS + glmnet), Boruta features (Boruta + glmnet) and plain glmnet. For each dataset, all three types of runs were performed 100 times with 100 different cross-validation seeds to assess the stability of the outcomes. (**B** and **C**) Performance of these models on the test sets. The plots on the second row (panel B) illustrate that there is no significant difference in the performance between the models that were built using features selected by SIVS and models that were built without despite the fact that the models built using SIVS use far fewer features as illustrated in panel A. Additionally, the plots in panel C illustrate the same data points as panel B, but are zoomed-in to show the performance robustness of models that are built using SIVS selected features compared to glmnet and Boruta + glmnet. (**D**) Venn diagrams depicting the overlap of the selected features via their intersection (∩) and union (∪), showing that the feature space suggested by SIVS is always a subset of standard glmnet feature space, and typically the feature space of SIVS is so robust that the intersect and union are the same set

**Fig. 3.**
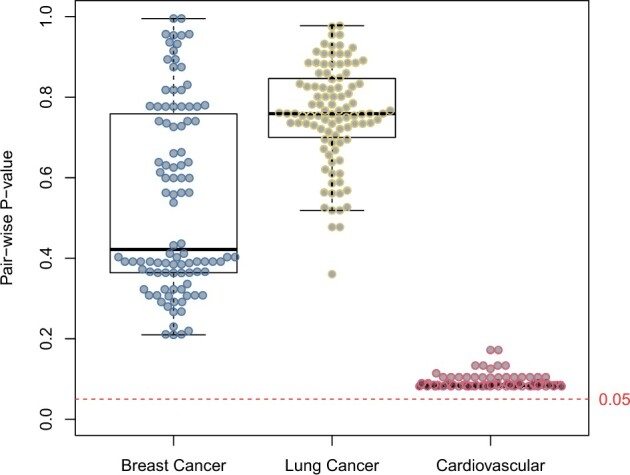
Significance of SIVS feature reduction on the final model. The AUROC of the glmnet models built using the full feature space and built using only SIVS suggested features were tested in a pair-wise fashion where models that were built using the same cross-validation seeds were compared together using the Delong method with two-sided alternative hypothesis (DeLong *et al.*, 1988)

For the breast cancer data, the standard glmnet models with median AUROC of 0.63 using the median of 76 features (range: 59–107) in the 100 different runs on the full feature space, whereas SIVS built models with median AUROC of 0.61 by constantly using 41 features (Table 2 and Fig. 2A–C). On lung cancer data, a median AUROC of 0.99 was achieved with the standard glmnet using the median of 114 features (range: 76–158), while with SIVS, the median AUROC of 0.99 was achieved using the median of 43 features (range: 41–45). On the cardiovascular disease data, the standard glmnet obtained a median AUROC of 0.70 using the median feature of 15 (range: 14–15), whereas with SIVS, the median AUROC of 0.69 was obtained using 13 features throughout all the 100 models. Thus, SIVS on average selected 49.6% fewer features compared to standard glmnet in all datasets, and on average 61.7% fewer features in high-dimensional datasets, while the models built using these features produced similar AUROC values (Table 2 and Fig. 2B). The number of features with SIVS was significantly lower than with standard glmnet (paired Wilcoxon test *P*-values 3.50e−18, 3.88e−18, and 4.71e−20, for breast cancer, lung cancer, and cardiovascular, respectively) without significantly affecting the AUROC values of the final models (DeLong median of *P*-values 0.42, 0.76, and 0.09, for breast cancer, lung cancer, and cardiovascular, respectively) (Fig. 3). By looking closer at the performances of each type of run, we see that while the models built based on features selected by SIVS use fewer features compared to their counterparts, their performance is more uniform across different runs with different cross-validation random seeds (Fig. 2C) which indicates the stability and robustness of the models built using features selected by SIVS. We observed similar behavior and performance on the Arcene benchmarking dataset as compared to the aforementioned results (Fig. 2 and Supplementary Table S2). Additional performance metrics for all datasets that show similar trends as AUROC are available in Supplementary Table S2.

**Table 2.** Run-type comparison and their models’ consistency

	Run-type	glmnet				SIVS + glmnet				Boruta + glmnet			
Metric	Detail	Breast cancer	Lung cancer	Cardiovascular	Arcene	Breast cancer	Lung cancer	Cardiovascular	Arcene	Breast cancer	Lung cancer	Cardiovascular	Arcene
Number of selected features	Maximum	107	158	15	59	41	45	13	10	7	35	11	7
	Median	76	114	15	43	41	43	13	10	7	34	11	6
	Mean	79.12	114.08	14.74	41.88	41	42.42	13	10	7	33.62	11	6.44
	Minimum	59	76	14	28	41	41	13	10	7	32	11	6
	Standard deviation	9.6685	15.9524	0.4408	7.9089	0	1.165	0	0	0	0.8261	0	0.4989
	Intersect	54	58	14	26	41	41	13	10	7	30	11	6
	Union	112	177	16	60	41	45	13	10	7	35	11	7
AUROC [validation]	Maximum	64.05%	99.36%	69.58%	75.53%	61.16%	99.37%	69.43%	72.73%	56.96%	99.28%	69.38%	69.48%
	Median	62.51%	99.19%	69.51%	74.84%	60.88%	99.30%	69.37%	71.39%	56.93%	99.16%	69.31%	69.32%
	Mean	62.01%	99.17%	69.52%	74.74%	60.93%	99.30%	69.37%	71.58%	56.93%	99.16%	69.32%	69.24%
	Minimum	59.42%	98.83%	69.45%	74.19%	60.74%	99.25%	69.37%	70.74%	56.89%	98.99%	69.31%	68.59%
	Standard deviation	0.0108	0.0009	0.0003	0.0041	0.001	0.0002	0.0001	0.0053	0.0002	0.0005	0.0002	0.0023

*Note*: For each data type that is used in this article and for each method, 100 modelings and testings have been done using 100 different cross-validation seeds. This table presents the consistency of each method in terms of the number of selected features and AUROC.

While SIVS produced a smaller feature space than the standard glmnet, the overlap of the selected features by SIVS across the different runs was markedly higher (Fig. 2D). In addition, the small standard deviation of the AUROC values across the 100 different runs, further supported the stability of the SIVS-based models. Moreover, it is worth noting that the features selected by SIVS were a subset of the features selected by the standard glmnet (Fig. 2D).

Comparisons with Boruta and RFE revealed that SIVS performed consistently as well as them or even slightly outperformed them in terms of the number of selected variables (Fig. 2 and Supplementary Fig. S1). Most importantly, SIVS produced substantially more stable feature sets compared to stock glmnet (Fig. 2 and Table 2) but also compared to RFE, especially in Arcene dataset (Supplementary Fig. S1).

## 4 Discussion

This study introduces SIVS, a novel feature selection method that can effectively reduce the feature space, especially in high-dimensional data, and provides insight into each feature's impact with regards to the response value. SIVS starts from aggregating the results of multiple multivariable modeling runs using different cross-validation random seeds. As a result, it provides feature importance scores for the features and consequently orders them accordingly. This score is then utilized in an RFE step to inspect the effect of each feature's removal on the stability and predictive power of the resulting model, which is ultimately used in narrowing down the list of important features to a much smaller subset.

To assess the performance of SIVS and the goodness of the selected features, 100 models built using SIVS’ features were compared with 100 models built using plain glmnet. These models were compared based on their predictive power on a separate test set, considering the number of features they have and the variability of these features among the 100 models. This procedure was applied to three different datasets. The presented results demonstrate the effectiveness of SIVS as a feature selection method on various high- and low-dimensional biomedical data, where SIVS reduced the feature space down to 38% of the features that LASSO can typically select, without having any significant drawback in the predictive power of the model over independent test sets. Moreover, the features selected by SIVS were markedly more stable over multiple runs than those selected by standard glmnet.

Feature selection methods can be divided into three categories: filter methods, wrapper methods, and embedded methods (Saeys *et al.*, 2007). In general, filter methods are independent of the machine learning method, i.e. model-agnostic, whereas wrapper and embedded methods are model-dependent. There have been some attempts to address model-agnostic feature selection on high-dimensional data (Labani *et al.*, 2018; Reggiani *et al.*, 2018; Yu and Liu, 2003), but the majority of available methods are model-dependent. Among these, some are designed to work for specific types of application (Wehrens and Franceschi, 2015), and some are more general (Perrot-Dockès *et al.*, 2017; Xu and Chen, 2014; Wehrens and Franceschi, 2015). SIVS is a method that falls into the model-dependent wrapper category, but due to the usage of internal methods, it is not dependent on one specific algorithm and is, therefore, more versatile. Although in the present study, we focused on glmnet as the underlying feature selection and model building method, the general concept can, in theory, be extended to methods with embedded feature selection/importance that depends on cross-validation for evaluating the weights for features such as random forests or Generalized Boosted Regression Models.

At the time of writing this article, there are 51 packages dependent on glmnet, out of which seven address feature selection [BioMark (Wehrens and Franceschi, 2015), elasso (Guo, 2015), EstHer (Bonnet and Levy-Leduc, 2015), glmvsd (Nan *et al.*, 2016), GRridge (van de Wiel and Novianti, 2020), MultiVarSel (Perrot-Dockès *et al.*, 2019) and SMLE (Zang *et al.*, 2021)]. As is suggested by our results, standard glmnet models were inherently not consistent in terms of the selected features or accuracy and, therefore, methods that use glmnet models internally without building multiple glmnet models and somehow aggregate their results are also susceptible to inherit this inconsistency.

SIVS is shipped with a method to suggest an appropriate cutoff for the exclusion of features with lower importance. The strictness of this suggestion method can be tuned (default = 0.01), and it is important to note that there is no one size fits all solution. The strictness threshold is subjective, and we encourage users to choose the threshold based on the RFE plot. To stay fair in this study, we consistently used the default parameters without modifications or tuning, but this is not to undermine the fact that the SIVS should not be treated as a blackbox feature selection method, and the parameters should be tuned according to the specification of data and the question in hand.

A major strength of this study is that we have focused on real-world datasets instead of synthetic data to demonstrate the practical utility of the method. Moreover, we have used independent validation datasets to show how much the selected features generalize to other datasets. Lastly, we provide a ready-to-use implementation of the method. The method is implemented in R language and in compliance with the Comprehensive R Archive Network (CRAN) standards and regulation and is published on CRAN. Therefore, SIVS can be freely accessed, installed, and tested. Additionally, the SIVS source code is published under General Public License v3.0 (GPL3) and is publicly available on Github.

A drawback of SIVS is that despite the multithreaded implementation of the method, it is relatively slow to compute due to multiple iterations (*k* = 100 by default). Additionally, considering that SIVS is wrapping the internal method, consequently it will inherit the limitation of that method as well (in the case of current implementation, glmnet). For example, due to L1 regularization, LASSO is known to have issues with colinear features which was not explored here. However, the aim of this study was to show the feasibility of the proposed method with a working implementation. For further optimization in the future, various possible alternatives could be considered. For example, the effect of replacing LASSO with a smooth function or exploring the effect of colinear features on SIVS performance as well as implementing alternative performance metrics and testing other prediction scenarios, such as multilabel classification, will be left as next steps for future research.

In this article, we have showcased SIVS with glmnet as an internal method via three binary classifications, but in theory, SIVS can be applied on any of the model families that glmnet can be used for, as long as the predicted outcome can be used in receiver operating characteristic (ROC) curve calculation in pROC package. This limitation could also be loosened by the addition of other performance metrics into SIVS. In addition to glmnet, SIVS also naturally extends to other forms of modeling with embedded feature selection or shrinkage methods, such as random forest. Implementing these additional internal methods and other performance metrics will be done in the next versions of the SIVS R package.

## 5 Conclusion

This study shows how a single run of glmnet is not an optimal solution for finding the best feature space in terms of consistency in performance and the number of incorporated features in the final model. SIVS, the method presented in this article, is a feature selection method that can drastically reduce the feature space without substantially sacrificing the performance and produces consistent results across multiple runs. This indicates that the ‘true signal’ is more effectively captured by SIVS compared to the standard glmnet.

All the scripts for data preprocessing and analysis are available upon request. The SIVS can be directly installed from CRAN, and the source code can be accessed through the following webpage:

- https://cran.r-project.org/package=sivs

## Author contributions

M.M. participated in the study design, conducted the analyses, developed the method, wrote the manuscript and developed the SIVS R package. M.S.V. participated in method development, participated in study design, preprocessed the clinical data, participated in the analyses and participated in writing the manuscript. R.K. participated in the development of the method, participated in study design and edited the manuscript. L.L.E. supervised the study, participated in the study design and participated in writing the manuscript.

## Supplementary Material

btab501_Supplementary_Data
