# Supplementary material for: Stable Iterative Variable Selection
Source: Bioinformatics. 2021 Jul 16;37(24):4810–7. doi: 10.1093/bioinformatics/btab501 (PMC8665768; doi:10.1093/bioinformatics/btab501)
Supplement: btab501_Supplementary_Data [file btab501_supplementary_data.zip › Appendix_merged.docx]

# Appendix

## Table S1 - List of all non-base R packages that have been used in this study

This table lists all the non-base R packages that have been used in this article sorted alphabetically based on their names. The second column, loaded versions, contains the exact version number of the R packages; and the last column, source, contains the repository that the packages were obtained from.

** The table is provided as a tabular data file (CSV) as instructed in the Author Instructions page of the journal **

Table S2 – Binary classification performance scores

All the calculated performance metrics used for the binary classification of single runs are collected in this table. For calculating the F1 and accuracy, the predicted class has been used, and for AUROC, the continuous predicted responses were used. As for Arcene data, considering that it had classes -1 and 1, the -1 was considered as class 0 for the F1 column in this table.

| **Dataset** | **Features Selection** | **Predicted Data** | **F1** | **Accuracy** | **AUROC** |
| --- | --- | --- | --- | --- | --- |
| **Breast Cancer** | **glmnet** | **train** | 0.9254 | 0.9476 | 0.9873 |
|  |  | **test** | 0.4366 | 0.5960 | 0.6251 |
|  | **SIVS** | **train** | 0.9671 | 0.9755 | 0.9972 |
|  |  | **test** | 0.4938 | 0.5859 | 0.6108 |
|  | **Boruta** | **train** | 0.6526 | 0.7692 | 0.8212 |
|  |  | **test** | 0.4459 | 0.5606 | 0.5693 |
| **Lung Cancer** | **glmnet** | **train** | 0.9911 | 0.9915 | 0.9997 |
|  |  | **test** | 0.9600 | 0.9600 | 0.9923 |
|  | **SIVS** | **train** | 0.9900 | 0.9905 | 0.9995 |
|  |  | **test** | 0.9608 | 0.9600 | 0.9930 |
|  | **Boruta** | **train** | 0.9362 | 0.9407 | 0.9897 |
|  |  | **test** | 0.9326 | 0.9350 | 0.9904 |
| **Cardiovascular** | **glmnet** | **train** | 0.0037 | 0.9382 | 0.7215 |
|  |  | **test** | 0.0000 | 0.9064 | 0.6951 |
|  | **SIVS** | **train** | 0.0147 | 0.9386 | 0.7213 |
|  |  | **test** | 0.0000 | 0.9061 | 0.6937 |
|  | **Boruta** | **train** | 0.0037 | 0.9381 | 0.7124 |
|  |  | **test** | 0.0000 | 0.9064 | 0.6941 |
| **Arcene** | **glmnet** | **train** | 1.0000 | 1.0000 | 1.0000 |
|  |  | **test** | 0.6437 | 0.6900 | 0.7488 |
|  | **SIVS** | **train** | 0.9767 | 0.9800 | 1.0000 |
|  |  | **test** | 0.6517 | 0.6900 | 0.7159 |
|  | **Boruta** | **train** | 0.8571 | 0.8700 | 0.9367 |
|  |  | **test** | 0.6429 | 0.7000 | 0.6944 |

##
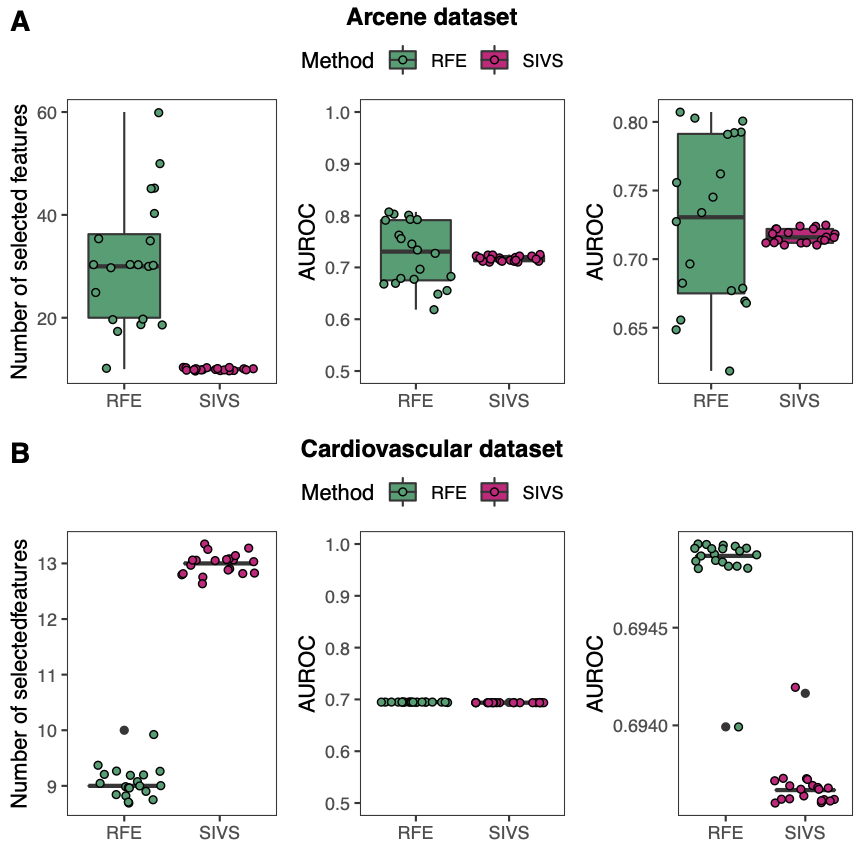


## Supplementary Figure S1 – Performance of SIVS against recursive feature elimination (RFE).

The number of features that were used in each of the 20 models built using glmnet with SIVS (SIVS + glmnet) and RFE, as well as their performances in terms of AUROC in A) Arcene and B) Cardiovascular datasets. RFE implementation in R package *caret* was used. RFE was run using 10-fold cross-validation and the final model returned during each run was used for predicting the binary outcomes.
