# Supplementary figures and images for: Stable Iterative Variable Selection
Source: Bioinformatics. 2021 Jul 16;37(24):4810–7. doi: 10.1093/bioinformatics/btab501 (PMC8665768; doi:10.1093/bioinformatics/btab501)

**A****Arcene dataset**Method 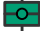 RFE 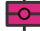 SIVS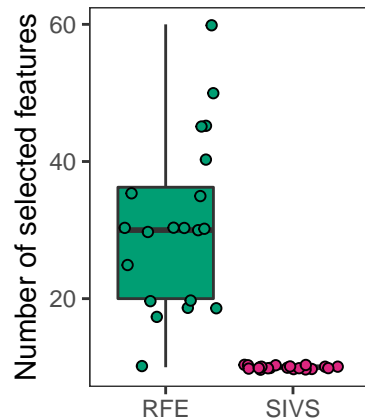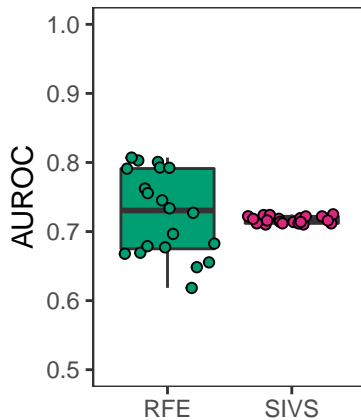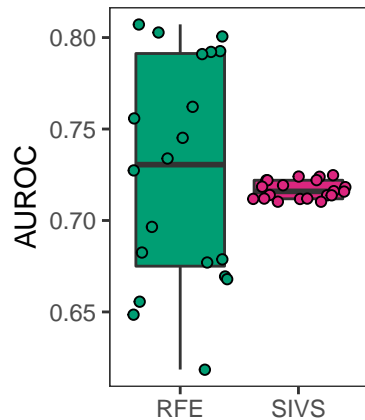**B****Cardiovascular dataset**Method 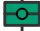 RFE 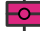 SIVS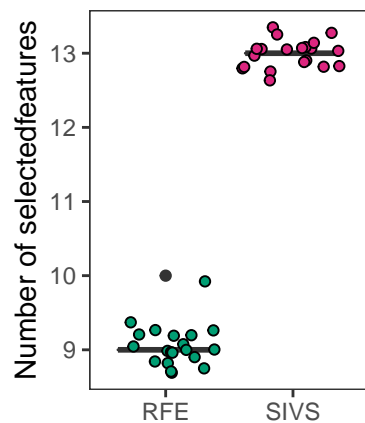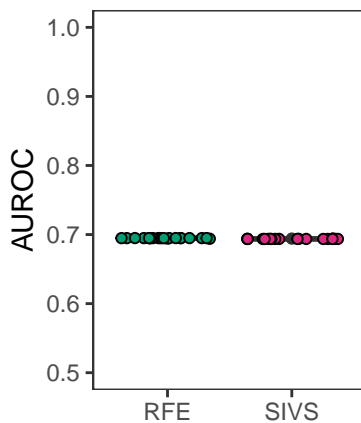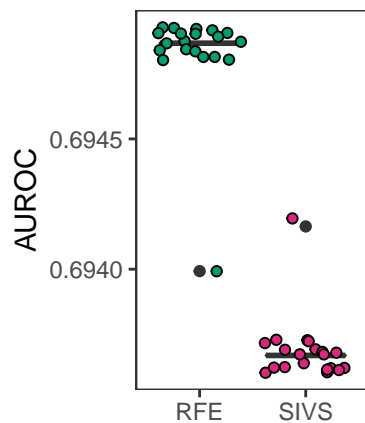

Supplement: btab501_Supplementary_Data [file btab501_supplementary_data.zip › supFigure1.pdf]
